# Supplementary material for: Structural basis for two-way communication between dynein and microtubules
Source: Nat Commun. 2020 Feb 25;11:1038. doi: 10.1038/s41467-020-14842-8 (PMC7042235; doi:10.1038/s41467-020-14842-8)
Supplement: Supplementary file 1 — Supplementary information [file 41467_2020_14842_MOESM1_ESM.pdf]

Supplementary information

Structural basis for two-way communication between dynein and microtubules

N. Nishida et al

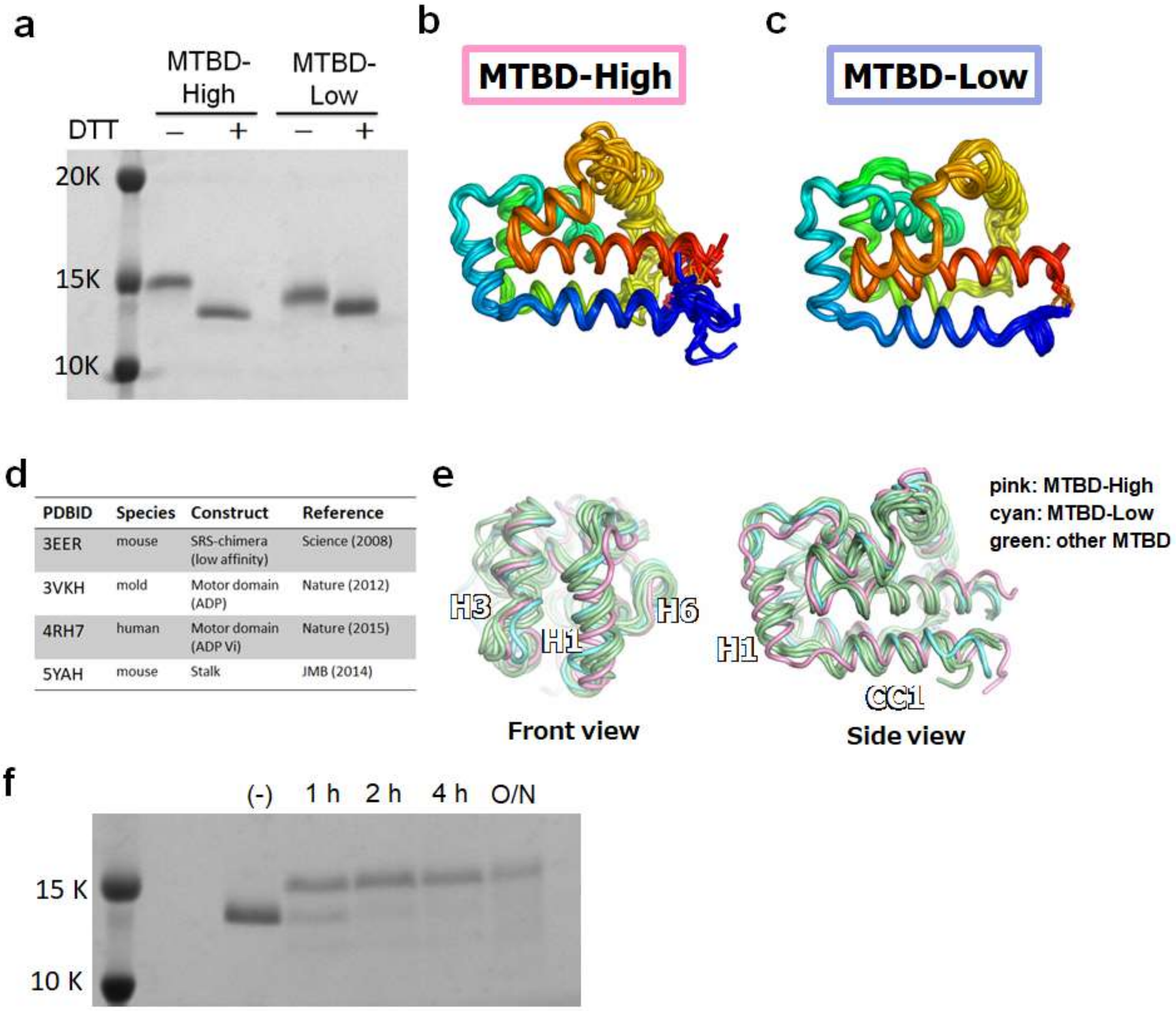

PDBID	Species	Construct	Reference
3EER	mouse	SRS-chimera (low affinity)	Science (2008)
3VKH	mold	Motor domain (ADP)	Nature (2012)
4RH7	human	Motor domain (ADP Vi)	Nature (2015)
5YAH	mouse	Stalk	JMB (2014)

Supplementary Figure 1.

The biochemical characterization and the NMR structures of MTBD-High and MTBD-Low.

(a) SDS-PAGE gel of MTBD-High and MTBD-Low. The samples were resuspended in the standard SDS loading buffer in the presence (+) and absence (-) of 10 mM DTT at 95 ºC for 5min, and then treated with 100 mM iodeactoamide (IAA). (b, c) NMR structures of MTBD-High and MTBD-Low. Backbone trace of the ten lowest-energy structures of the MTBD-High (b) and MTBD-Low (c) structures. NMR structure and refinement statistics are provided in Supplementary Table 1 (d, e) Comparison of the NMR structures of MTBD-High and MTBD-Low with previously reported crystal structures of MTBD. (d) A table summarizing the previously determined crystal structures of dyneins containing the MTBD moiety[1–4]. (e) Superposition of MTBD-Low (cyan), MTBD-High (pink), and the MTBD moiety of the previous crystal structures (light green). (f) Confirmation of disulfide bond reduction of MTBD-High by 1 mM DTT treatment at room temperature. The samples were treated at room temperature in the absence (-) or presence of 1 mM DTT for 1, 2, or 4 hours or overnight (O/N), and then treated with 50 mM IAA. Uncropped images of SDS-PAGE gels are provided as a Source Date file.

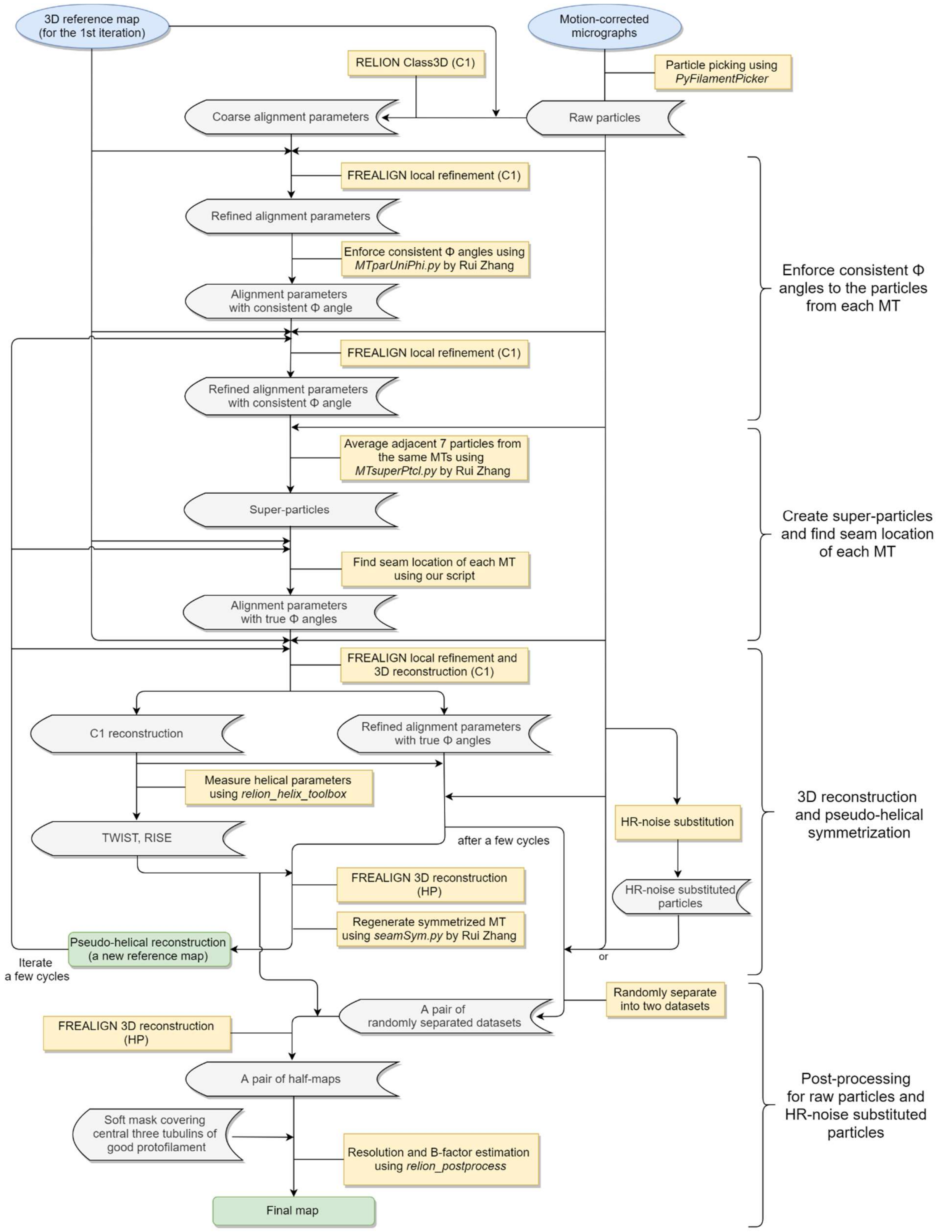

Note: Three-dimensional (3D) reconstructions using RELION or FREALIGN are performed either in C1 (asymmetric) or HP (pseudo-helical; implemented only in FREALIGN) reconstruction mode.

Supplementary Figure 2.

Processing workflow for the cryo-EM structures of MTBD-High-MT complexes

Seam determination was performed using the "super-particle"-based approach with a few modifications using original scripts developed in our lab. In the post-processing steps, Fourier Shell Correlation (FSC) calculation and

B-factor estimation were performed using *relion_postprocess* with a soft mask covering the central three tubulins of one “good” protofilament. To calculate FSC_{true}, we calculated both FSC_t from the raw dataset and FSC_n from the high-resolution (HR) noise-substituted dataset.

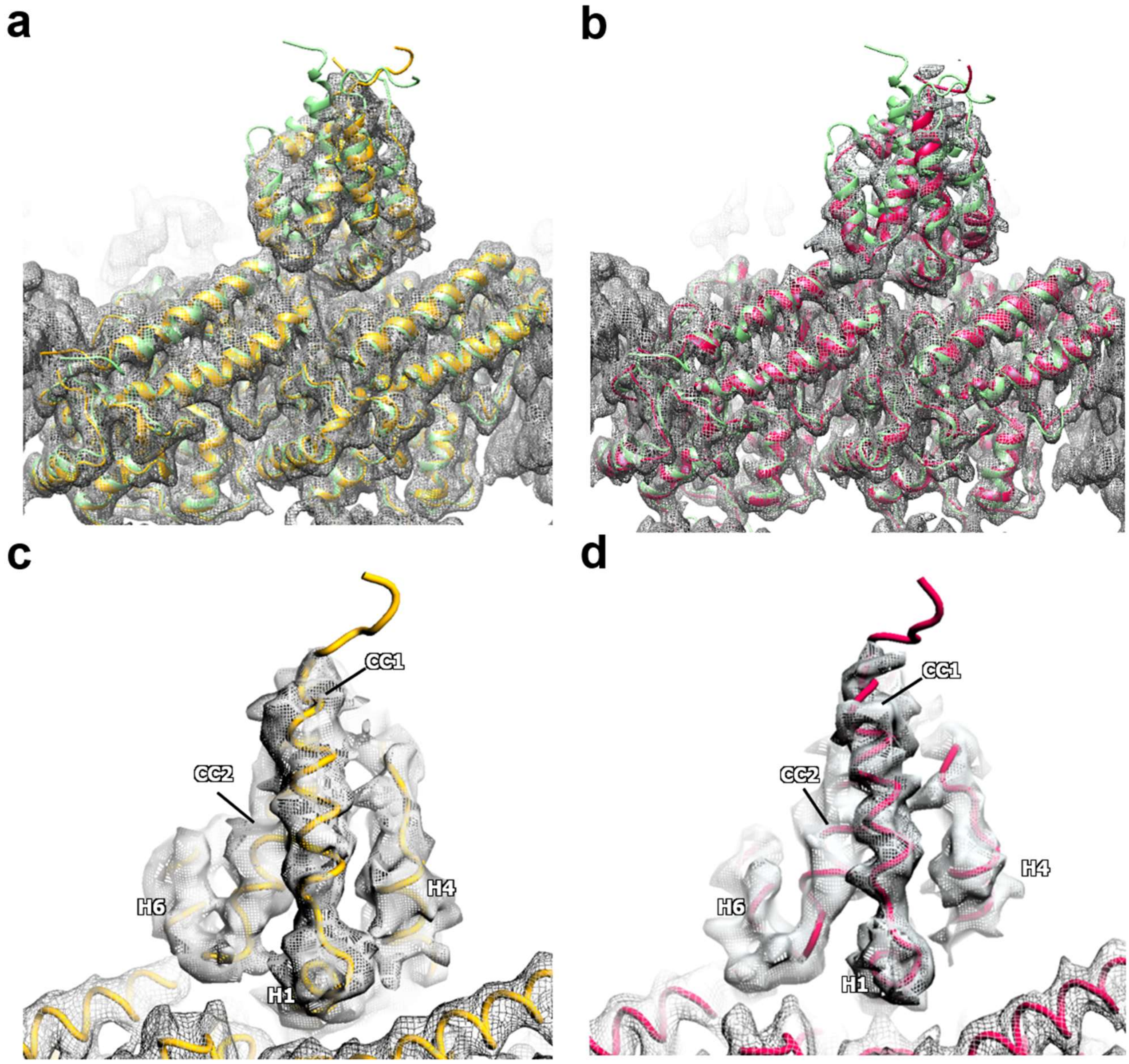

Supplementary Figure 3

Flexible fitting of MTBD-High and tubulin dimer into the cryo-EM maps of MTBD-High-MT complex in the absence (-) and presence (+) of DTT.

(a, b) The molecular dynamics flexible fitting (MDFF) of MTBD-High and the tubulin dimer (PDB code: 1JFF) into the cryo-EM maps filtered to 4 Å: (a) for DTT(-) and (b) for DTT(+).The initial placement of the complex is shown in light green. (c, d) Close-up views of the CC1 moiety fit into the cryo-EM maps filtered to 4 Å: (c) for DTT(-) and (d) for DTT(+).

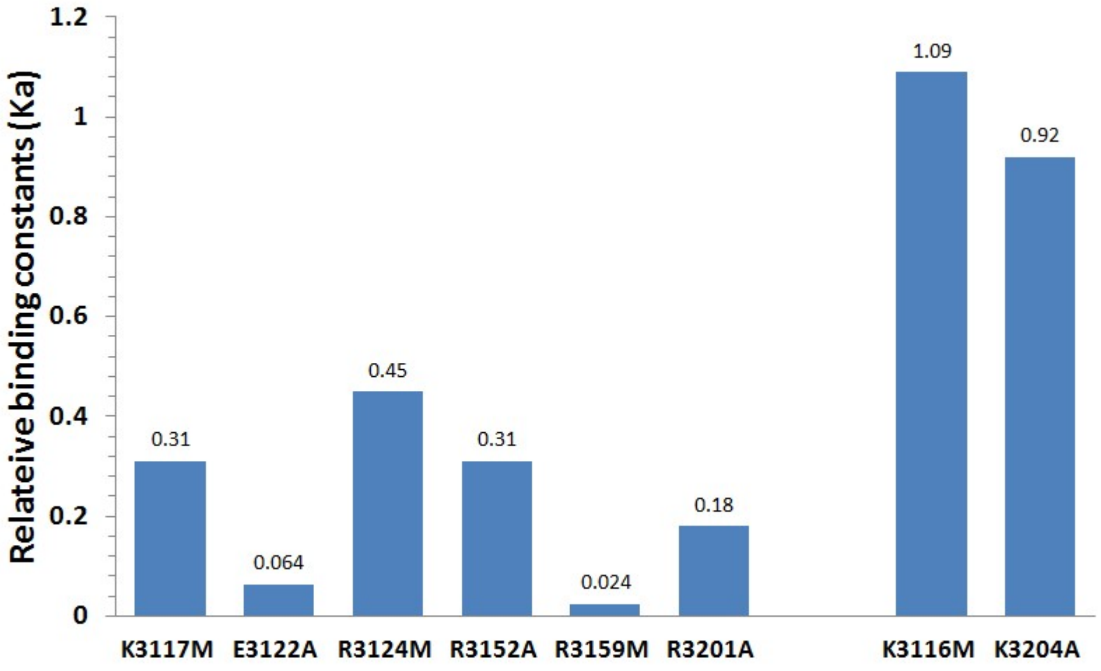

Supplementary Figure 4.

Mutational analysis of MTBD-High.

The binding constants (normalized to those of MTBD-High) for each mutant are plotted as a bar graph. We confirmed that the overall conformation of MTBD was not disrupted either the mutation based on the HSQC (heteronuclear single quantum coherence) spectra of the mutants.

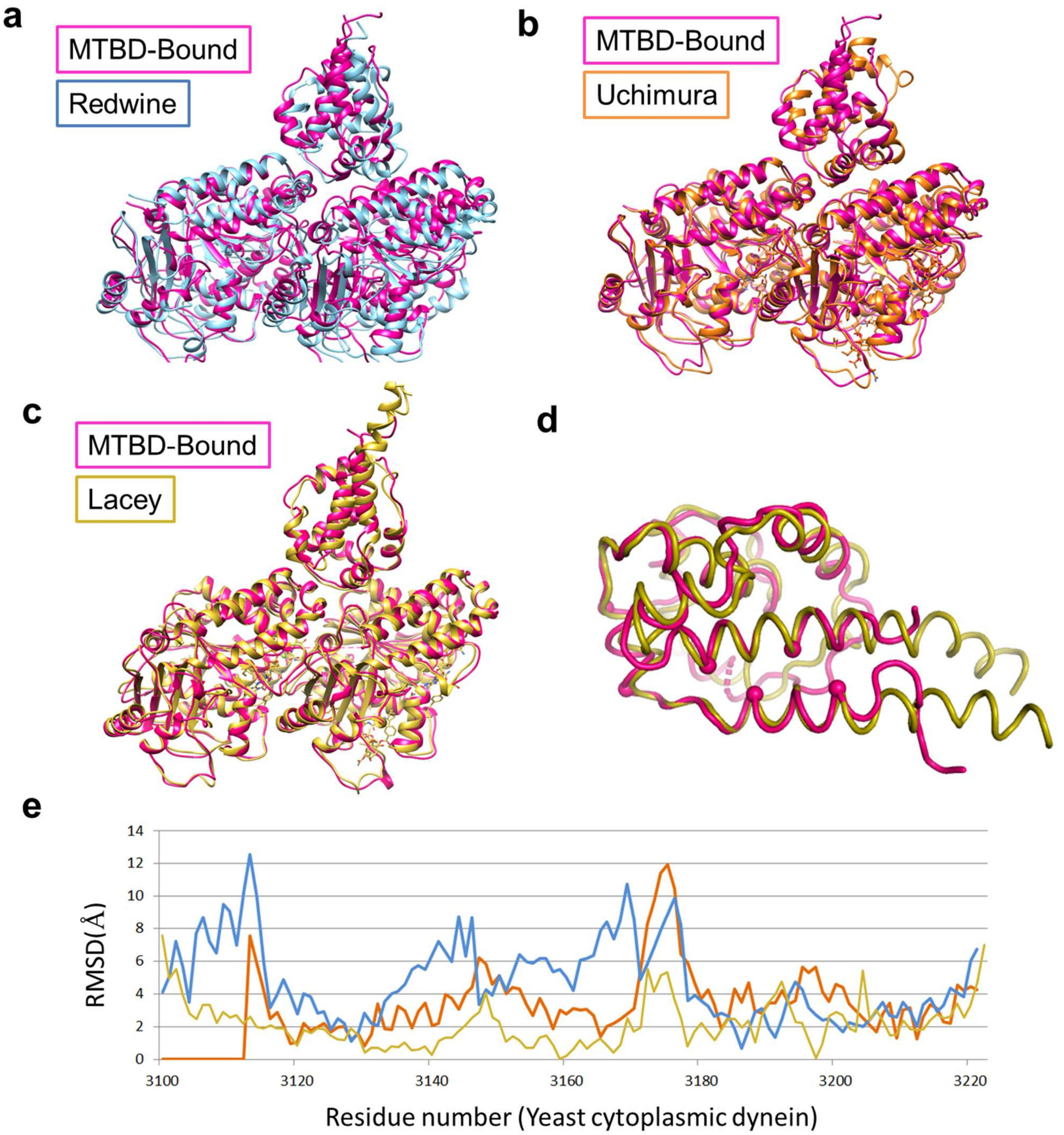

Supplementary Figure 5.

Comparison of overall structure of three cryo-EM-based models of dynein MTBD-MT complexes.

(a-c) Superimposition of the cryo-EM based models of (a) Redwine et al.[5] (cyan), (b) Uchimura et al.[6] (orange), and (c) Lacey et al.[7] (yellow) with the model derived in the current study (magenta). All structures are aligned with respect to the α-tubulin subunit. (d) Superposition of MTBD moiety between the Lacey model (yellow) and that of the present study (magenta). (e) Plots of RMSD values for the Cα position of each MTBD residue in the current model and in those of the Redwine (cyan), Uchimura (orange), and Lacey (yellow) models. Source data are provided as a Source Data File.

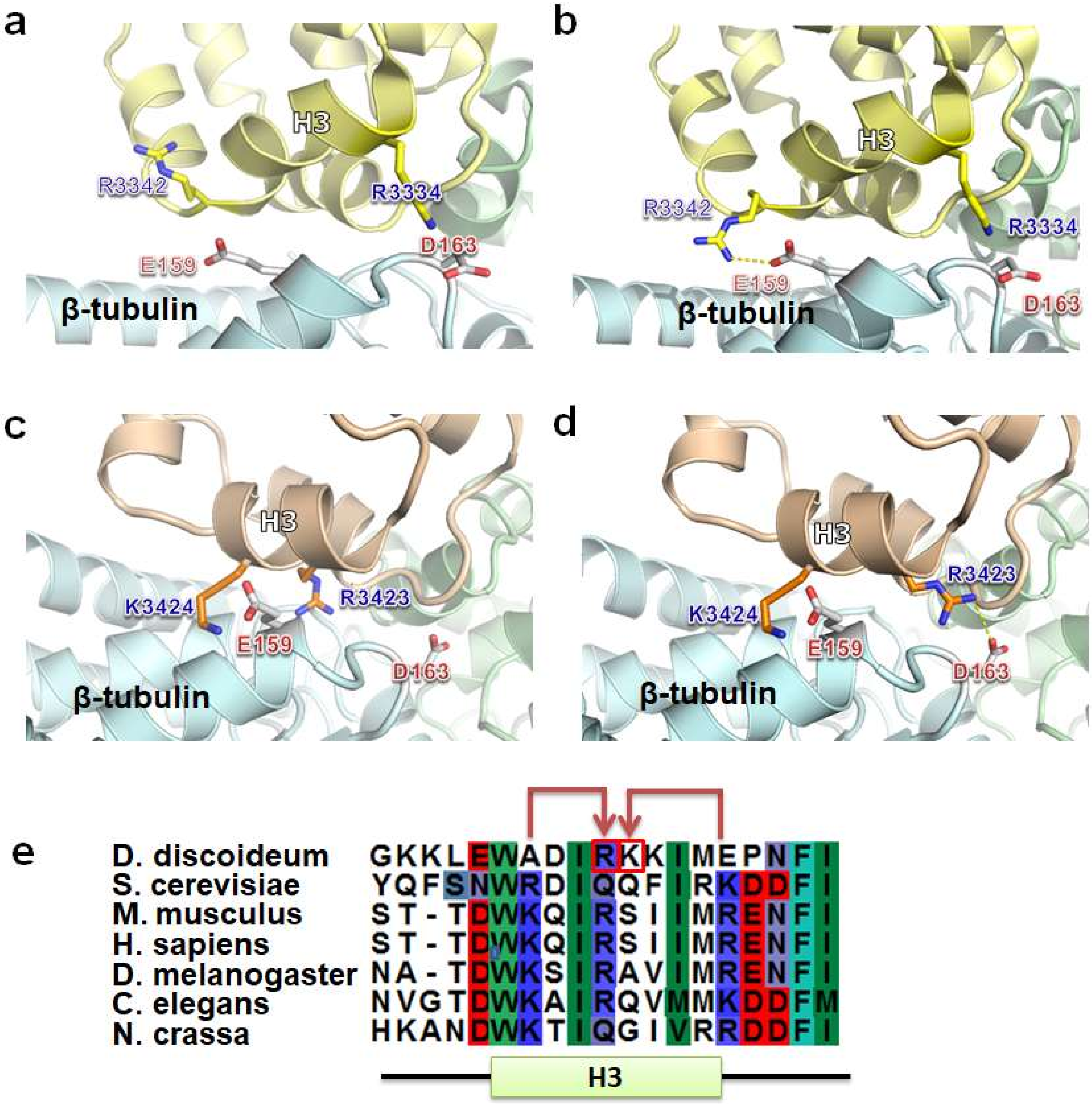

Supplementary Figure 6.

Conservation of the interaction mode of H3.

(a) The conserved salt bridge forming H3 residues of mouse cytoplasmic dynein are shown by stick models. (b) The manipulated side chain of R3342 re-orients toward E159, which is sufficiently close to form a salt bridge. (c) The conserved salt bridge-forming H3 residues of *Dictyosterium* cytoplasmic dynein are shown by stick models. (d) The manipulated side chain of R3423 re-orients toward D163, which is sufficiently close to form a salt bridge. (e) Sequence alignments of the H3 region of the cytoplasmic dyneins of various species.

Supplementary Table 1.

NMR and refinement statistics for protein structures.

	MTBD-High	MTBD-Low
**NMR distance and dihedral constraints**		
Distance constraints		
Total NOE	1518	1745
Shor-range ($\|i-j\| \leq 1$)	677	802
Medium-range ($2 \leq \|i-j\| \leq 4$)	407	440
Long-range ($\|i-j\| \geq 5$)	434	503
Hydrogen bonds	20	30
Total dihedral angle restraints		
ϕ	118	144
ψ	70	102
Residual dipolar coupling	75	100
**Structure statistics**		
Violations (mean and s.d.)		
Distance constraints (Å)	0.07 ± 0.004	0.063 ± 0.005
Dihedral angle constraints (º)	1.594 ± 0.308	0.977 ± 0.291
Max. dihedral angle violation (º)	16.225	17.298
Max. distance constraint violation (Å)	1.222	1.214
Deviations from idealized geometry		
Bond lengths (Å)	0.004 ± 0.000	0.004 ± 0.000
Bond angles (º)	0.576 ± 0.023	0.493 ± 0.032
Impropers (º)	0.599 ± 0.022	0.510 ± 0.039
Average pairwise r.m.s. deviation** (Å)		
Heavy	1.66 ± 0.19	1.48 ± 0.22
Backbone	0.96 ± 0.15	0.56 ± 0.08

**Pairwise r.m.s. deviation was calculated with residue 3101-3170 and 3180-3222 of ten refined structures.

Supplementary Table 2.

Cryo-EM data collection, processing and validation statistics.

Source data are provided as a Source Data file.

	#1 MTBD-MT DTT(-) (EMDB-9996) (PDB 6KIO)	#2 MTBD-MT DTT(+) (EMDB-9997) (PDB 6KIQ)
**Data collection and processing**		
Magnification	105k ×	105k ×
Voltage (kV)	200	200
Electron exposure (e–/Å^2)	54	54
Defocus range (μm)	-1.0 to -2.5	-1.0 to -2.5
Pixel size (Å)	1.32	1.32
Symmetry imposed	C1 and HP	C1 and HP
Initial MT filaments (no.)	2,479	1,427
Final MT filaments (no.)	1,920	1,044
Initial particle images (no.)	76,377	35,636
Final particle images (no.)	58,999	32,666
Map resolutions (Å)		
FSC threshold	0.143	0.143
C1 (asymmetric)		
FSC_{true} / FSC_t / FSC_n	4.4 / 4.5 / 8.8	4.3 / 4.3 / 9.1
HP (pseudo-helical)		
FSC_{true} / FSC_t / FSC_n	3.9 / 3.9 / 7.8	3.7/ 3.6 / 8.0
Map resolution range (Å)		
HP (pseudo-helical)	3.8 to 5.6	3.5 to 5.5
Map sharpening *B* factors (Å^2)		
C1 (asymmetric)	-91.3	-82.0
HP (pseudo-helical)	-145.0	-128.9
**Model validation**		
Initial model used (PDB code)	1JFF + 6KJN	1JFF + 6KJN
Backbone RMSD		
with respect to the initial model (Å)	1.96	1.91
Ramachandran plot		
Favored / Allowed / Disallowed (%)	81.3 / 15.1 / 3.6	77.2 / 17.0 / 5.8
CCC between map and model	0.67	0.67

Supplementary Table 3.

Primer sequences.

MTBD_WT_fwd	GCGCATATG AAAAGTATTCAAGATATCGAACCTAC
MTBD_WT_rev	CGCGGATCCTCATTCTTGTCTCAGCGGATCTACGTT
S3097C_fwd	GCGGCAGCCATATGAAATGTATTCAAGATATCGAACC
S3097C_rev	GGTTCGATATCTTGAATACATTTCATATGGCTGCCGC
I3101C_fwd	CCATATGAAAAGTATTCAAGATTGCGAACCTACCATTTTAGAGGCAC
I3101C_rev	GTGCCTCTAAAATGGTAGGTTCGCAATCTTGAATACTTTTCATATGG
V3222C_fwd	GCCCAAATAAACTTCAGTAAATGTTTAGAAAACGTAGATCCGCTGAG
V3222C_rev	CTCAGCGGATCTACGTTTTCTAAACATTTACTGAAGTTTATTTGGGC

Supplementary references

1. Carter, A. P. *et al.* Structure and functional role of dynein's microtubule-binding domain. *Science* **322**, 1691–1695 (2008).
2. Kon, T. *et al.* The 2.8 Å crystal structure of the dynein motor domain. *Nature* **484**, 345–350 (2012).
3. Schmidt, H., Zalyte, R., Urnavicius, L. & Carter, A. P. Structure of human cytoplasmic dynein-2 primed for its power stroke. *Nature* **518**, 435–438 (2015).
4. Nishikawa, Y. *et al.* Structure of the entire stalk region of the Dynein motor domain. *J. Mol. Biol.* **426**, 3232–3245 (2014).
5. Redwine, W. B. *et al.* Structural basis for microtubule binding and release by dynein. *Science* **337**, 1532–1536 (2012).
6. Uchimura, S. *et al.* A flipped ion pair at the dynein-microtubule interface is critical for dynein motility and ATPase activation. *J. Cell Biol.* **208**, 211–222 (2015).
7. Lacey, S. E., He, S., Scheres, S. H. & Carter, A. P. Cryo-EM of dynein microtubule-binding domains shows how an axonemal dynein distorts the microtubule. *Elife* **8**, e47145 (2019).
