# Supplementary material for: Structural basis for two-way communication between dynein and microtubules
Source: Nat Commun. 2020 Feb 25;11:1038. doi: 10.1038/s41467-020-14842-8 (PMC7042235; doi:10.1038/s41467-020-14842-8)
Supplement: Supplementary file 4 — Description of Additional Supplementary Files [file 41467_2020_14842_MOESM4_ESM.pdf]

Title: Supplementary Movie 1

Continuous movements of CC1 and H1 from MTBD-Low to MTBD-High to MTBD-Bound.

Description: A morphed series showing the conformational transition of the MTBD from MTBD-Low to MTBD-High to MTBD-Bound. Orientation and coloring are the same as those used in Figure 2d.
